# Non-Classical H1-like PARP1 Binding to Chromatosome

**DOI:** 10.3390/cells14171309

**Published:** 2025-08-25

**Authors:** Daria Koshkina, Natalya Maluchenko, Dmitry Nilov, Alexander Lyubitelev, Anna Korovina, Sergey Pushkarev, Grigoriy Armeev, Mikhail Kirpichnikov, Vasily Studitsky, Alexey Feofanov

**Affiliations:** 1 Faculty of Biology, Lomonosov Moscow State University, Moscow 119234, Russiaavfeofanov@yandex.ru (A.F.); 2 Belozersky Institute of Physicochemical Biology, Lomonosov Moscow State University, Moscow 119234, Russia; nilovdm@gmail.com (D.N.);; 3 Faculty of Bioengineering and Bioinformatics, Lomonosov Moscow State University, Moscow 119234, Russia; 4 Shemyakin-Ovchinnikov Institute of Bioorganic Chemistry, Russian Academy of Sciences, Moscow 117997, Russia; 5 Fox Chase Cancer Centre, Philadelphia, PA 19111-2497, USA

**Keywords:** PARP1, H1.0, nucleosome, PARylation, spFRET, EMSA, molecular modeling

## Abstract

Poly(ADP-ribose)polymerase 1 (PARP1) is an enzyme that interacts with chromatin during DNA repair and transcription processes; the molecular mechanisms of these processes remain to be determined. Previously, we have shown that PARP1 can bind to and reorganize nucleosomes using two modes of interaction with a mono-nucleosome, which are realized through PARP1 binding to the ends of linker DNA and to the nucleosome core. Here, it is shown that the latter mode of binding induces the reorganization of nucleosome structure and is more stable under the conditions of poly(ADP-ribosyl)ation (PARylation). The initial nucleosome structure is fully recovered after the dissociation of autoPARylated PARP1. The competition between PARP1 and linker histone H1.0 for binding to a nucleosome is mediated by the PARP1-H1.0 interaction and is affected by the length of linker DNA fragments. Longer linkers stabilize H1.0-nucleosome complexes, while shorter linkers facilitate displacement of H1.0 from the chromatosome by PARP1. PARylation removes both H1.0 and PARP1 from the complexes with nucleosomes. The data suggest that the H1.0 displacement from chromatin by PARP1 that is likely modulated by the density of nucleosomes might reduce chromatin compaction and facilitate access of PARP1-dependent DNA repair and transcription factors to nucleosome and inter-nucleosomal DNA.

## 1. Introduction

Poly(ADP-ribose)polymerase 1 (PARP1) is a nuclear protein that participates in DNA repair, replication, transcription, cell cycle regulation, and programmed cell death via enzymatic and non-enzymatic mechanisms [1,2,3,4,5]. The functioning of PARP1 in DNA repair and transcription processes is primarily related to its interaction with either damaged or intact DNA. PARP1 can recognize various types of DNA damage and has a high affinity for single- and double-strand DNA breaks (DSBs) [6,7,8]. Binding to DNA lesions activates PARP1 and leads to the NAD^+^-dependent synthesis of poly(ADP-ribose) (PAR) chains attached to nearby proteins and to PARP1 itself [4,5]. PARP1 is responsible for the majority (approximately 85–90%) of the poly(ADP-ribose) synthesis in cells [9,10]. PAR chains act as a molecular alarm signal to recruit repair factors (e.g., XRCC1, DNA ligases, and polymerases) to the damage site. AutoPARylation of PARP1 results in an accumulation of a negative charge and dissociation of the enzyme from DNA and its inactivation. PARP1 binds to intact DNA in an enzymatically inactive state with relatively low affinity [11]. This interaction is thought to accelerate the detection of DNA lesions by PARP1 [12].

The interaction of PARP1 with transcription factors is one way in which PARP1 regulates transcription [13,14,15]. Thus, in cortical neurons and cardiomyocytes, the activation of PARP1 enhances transcription of the gene *c-fos* through the PARP1-regulated phosphorylation of the transcription factor Elk1 [16]. PARP1 was found to interact with and to be a coactivator of inducible transcription factor NF-kappa B [17]. PARP1 was shown to be a cofactor of transcription factors Oct4 and Sox2 in their regulation of embryonic stem cell differentiation [18]. In a broader sense, PARP1 participates in regulating the DNA accessibility to various nuclear factors within chromatin, which is essential for both normal cell function and the response to stress conditions, in particular, ones associated with DNA damage [14].

Nucleosomes and chromatosomes are the basic structural units of chromatin, which can restrict the accessibility of DNA and create a barrier to transcription [19,20,21,22]. They also modulate the efficiency of DNA repair [23,24,25]. PARP1 together with other chromatin-binding factors dynamically modulates the efficiency of these restrictions and the height of barriers in a competitive manner. In vitro, PARP1 preferably binds to nucleosomes compared to the regions of intact DNA and can compete for the binding with linker histone H1 [5], thus preventing the assembly of nucleosomes into higher-order chromatin structures. In agreement with this observation, regions of PARP1 binding were found to be depleted of H1 in vivo [13,26]. The displacement of linker histone weakens inter-nucleosome interactions, which, in turn, can facilitate the repositioning of nucleosomes and affect the regulation of gene activity. Binding to nucleosomes activates catalytic activity of PARP1 [5] that could serve as the mechanism of chromatin reorganization through PARylation of chromatin proteins followed by their dissociation.

Association of PARP1 with active promoters observed in vivo [13,26,27] may indicate that PARP1 helps to displace nucleosomes from these promoters and attract transcription factors to them. In vitro, PARP1 reorganizes the structure of nucleosomal DNA [28], and in the presence of NAD^+^, positively regulates transcription, which is accompanied by the displacement of core histones from nucleosomal DNA [29]. In vivo, activated PARP1 was supposed to participate in removal of chromatin proteins from chromosomes, making them more available for remodeling and transcription [30]. These data suggest that, in addition to the “classical” mode of high-affinity interaction between PARP1 and damaged DNA and the low-affinity binding of PARP1 to intact DNA, there is also a “non-classical” mode of PARP1 interaction with nucleosomes and chromatosomes. This “non-classical” interaction appears to be important for transcription and DNA repair.

In this study, the two modes of interaction between PARP1 and mono-nucleosomes containing two DNA linkers were studied in the absence and presence of linker histone H1.0. It was found that PARP1 can compete with the linker histone bound to a nucleosome, and that this competition depends on the length of the DNA linkers. These observations help to understand the role played by PARP1 in the dynamic regulation of DNA accessibility within chromatin.

## 2. Materials and Methods

### 2.1. DNA Templates

Four DNA templates with length 187 bp that contain nucleosome positioning sequence (NPS) 603-42A, differently labeled with a pair of fluorescent labels Cy3 and Cy5, were obtained by polymerase chain reaction (PCR), as published earlier [31].

To produce the DNA template 187 bp labeled at positions 18 bp before and after NPS, the following fluorescently labeled oligonucleotides (Lumiprobe, Moscow, Russia) were used:

5′–CA[Cy3-dT]GCGACACCGGCACTGGGCCCGGTTCGCG

CGCCCGCCTTCCGTGTGTTGT–3′

5′–GA[Cy5-dT]CCATGATGGGCACTGGGTACCCCAGGGACTTG

AAGTAATAAGGAC–3′

To produce the DNA template 227 bp labeled at positions 25 bp before and after NPS, PCR was performed as described previously [32] using the following fluorescently labeled primers (Lumiprobe, Russia):

5′–ACACGGCGCACTGCC[Cy3-dT]ACCCAAACGACACCGGCACGAG–3′

5′–TAAGGCGAATTCAC[Cy5-dT]ACTTTCTGGCAAGAAAATGAGCT–3′

PCR products were purified in 2% agarose gel and extracted by QIAquick Gel Extraction Kit (Qiagen GmbH, Hilden, Germany) following the manufacturer’s protocol.

### 2.2. Protein Purification

Human recombinant PARP1 was expressed in *E. coli* and purified as described [33,34]. Full-size recombinant H1.0 histone *X. laevis* (wild-type) was obtained as described [35,36] using the *E. coli* strain kindly gifted by Prof. Dimitrov.

The pET3 plasmid containing gene of *X. laevis* H1.0 with G101C substitution was kindly provided by Prof. J.J. Hayes. Expression, purification, and modification of H1.0 were performed as described earlier [37]. *E. coli* Rosetta2-pLysS (DE3) cells were transformed with the plasmid and grown in the lysogeny broth medium containing 100 µg/mL ampicillin at 37 °C until the optical density of ~0.6 at 600 nm was reached. Protein expression was induced by adding isopropyl β-D-1-thiogalactopyranoside (0.5 mM), and, after 4 h of incubation at 37 °C, cells were harvested by centrifugation. Pelleted cells were resuspended in TE buffer (1 mM EDTA, 10 mM Tris-HCl, pH 8.0) containing 3.6 mM phenylmethylsulphonyl fluoride, 0.5 mg/mL lysozyme, 0.2% Triton X-100, 2 mM dithiothreitol (DTT), and 1 tablet of complete protease inhibitor cocktail (Roche), and incubated for 30 min at room temperature. After adjusting NaCl concentration to 1 M, the cell suspension was sonicated and centrifugated at 11,000× *g* for 45 min. Supernatant was collected, diluted to the 0.5 M NaCl concentration, and incubated with 1.5 mL of BioRex 70 resin (Bio-Rad, Hercules, CA, USA) for 4 h at 40 °C. Resin loaded with linker histone protein was collected in a single-use chromatography column, washed with TE buffer containing 0.6 M NaCl (15 mL) and with TE buffer containing 0.7 M NaCl (5 mL). Protein was then eluted with TE buffer containing 2 M NaCl (10 mL) as 1 mL fractions. Protein-containing fractions were collected, combined, and diluted to 0.5 M NaCl concentration. Concentration and purity of target protein was assessed by SDS-PAGE.

Protein solution was then incubated with 1 mL of BioRex 70 resin for 4 h, and its elution from the chromatographic column was performed with TE buffer containing 0.6 M NaCl (3 mL), 0.7 M NaCl (0.5 mL), and 2 M NaCl (2 mL). To remove residual nucleic acid, protein solution was incubated with 5 mL of hydroxyapatite for 1 h at 40 °C and then centrifugated at 11,000× *g* for 30 min.

Prior to maleimide conjugation, the protein was treated with 50 mM DTT for 1 h at room temperature to reduce disulfide bonds. Reduced protein was purified with BioRex 70 resin using elution with TE buffer containing 0.6 M NaCl (20 mL), 0.7 M NaCl (5 mL), and 2 M NaCl (5 mL) as described above. Collected fractions were immediately shock-frozen with liquid nitrogen.

Modification of protein with sulfo-Cy5 was performed by incubation of protein solution with 8× molar excess of sulfo-Cy5-maleimide (Lumiprobe, Russia) for 30 min at room temperature in the dark. The reaction was terminated by adding DTT to a final concentration of 10 mM. Cy5-modified H1.0 was then purified with BioRex 70 resin as described above using TE buffer containing 0.6 M NaCl (50 mL), 0.7 M NaCl (5 mL), and 10 mL of 2 M NaCl (10 mL) for washing and elution.

### 2.3. Assembly of Nucleosomes

**Figure 1:**
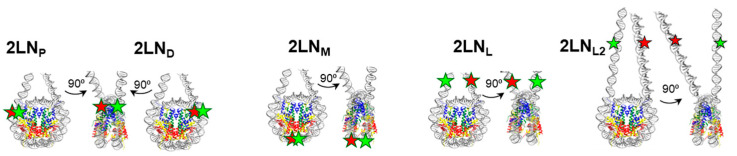
The positions of the donor–acceptor FRET pairs of Cy3/Cy5 labels within the nucleosomes. 2LN nucleosomes were assembled on 187 bp (2LN_P_, 2LN_M_, 2LN_D_, 2LN_L_) or 227 bp (2LN_L2_) DNA templates containing centrally localized nucleosome positioning sequence 603-42A and two adjacent 20 bp or 40 bp linker DNA fragments. Nucleosomes contained a pair of fluorescent labels (Cy3 and Cy5, green and red asterisks, respectively).

Nucleosomes were assembled using fluorescently labeled DNA templates and chicken-donor chromatin without linker histone, as described earlier [38]. A panel of two-linker nucleosomes (2LN) with fluorescent labels in different regions of nucleosomal DNA was used (Figure 1).

To study the structural rearrangements in the core region of a nucleosome, fluorescent labels Cy3 and Cy5 were attached to thymine bases at positions 13 and 91 bp (2LN_P_), 35 and 112 bp (2LN_M_), or 57 and 135 bp (2LN_D_) from the beginning of NPS. After assembly of nucleosomes, Cy3 and Cy5 become located on adjacent gyres of nucleosomal DNA and support efficient Förster resonance energy transfer (FRET). The positions of the labels were selected to provide orientation of labels in the solution and to avoid interfering with the interactions between DNA and core histones. 2LN_P_ and 2LN_D_ were designed to probe structural changes occurring on both sides near the nucleosome boundary. 2LN_M_ was created to study the alterations in structure that occur deep inside the nucleosome. 2LN_L_ nucleosomes (DNA length is 187 bp) were assembled with the Cy3 and Cy5 labels positioned in linker DNA fragments (20 bp each linker) at a distance of 18 bp before and after NPS. 2LN_L2_ nucleosomes (DNA length is 227 bp) were assembled with the Cy3 and Cy5 labels positioned in linker DNA (40 bp each linker) at a distance of 25 bp before and after NPS (Figure 1). 2LN_L_ and 2LN_L2_ were designed to probe structural changes in the linker DNA region.

Assembled nucleosomes were analyzed by the electrophoresis in the 4.5% polyacrylamide gel (acrylamide: bisacrylamide 39:1; 0.5× TBE buffer). TBE buffer contained 90 mM Tris-borate and 2 mM EDTA (pH 8.3). Assembled nucleosomes were isolated from the corresponding region of the gel by extraction in a buffer containing 10 mM HEPES-NaOH (pH 8.0), 0.2 mM EDTA, and 0.2 g/L bovine serum albumin, and stored at 4 °C. According to electrophoresis data, content of free DNA in the purified nucleosomes was less than 5%.

### 2.4. Sample Preparations

All the experiments were performed in a TB150 buffer (20 mM Tris-HCl, 5 mM MgCl_2_, 150 mM KCl, 1 mM β-mercaptoethanol, pH 7.9). Concentrations of nucleosomes were 1 and 2–3 nM in the samples for spFRET and EMSA experiments, respectively. Fluorescently labeled nucleosomes were incubated for 30 min at 25 °C in the presence of different concentrations of PARP1 in low-adhesion tubes. To induce polyADP- ribosylation (PARylation), nucleosomes were pre-incubated with PARP1 (40 nM) for 15 min, mixed with NAD^+^ (100 μM), and incubated for 35–40 min. Chromatosomes were formed by incubating nucleosomes with histone H1.0 (5 or 10 nM) for 20 min at 25 °C in TB150 buffer. In the competitive binding experiment, H1.0 (10 nM) was incubated with nucleosomes for 20 min, and then PARP1 (40 nM) was added and followed by 30 min incubation of the mixture. When necessary, NAD^+^ (100 μM) was added to this sample 15 min later, followed by a 35–40 min incubation of the reaction mixture.

### 2.5. SpFRET Experiments

spFRET measurements were performed as described in [39]. spFRET microscopy was performed in highly diluted solutions of nucleosomes, measuring FRET from single particles (nucleosomes or their complexes), which diffused through the laser beam focus. The proximity ratio (E_PR_) was calculated for each measured particle:

E_PR_ = (I_5_ − 0.19 × I_3_)/(I_5_+ 0.81 × I_3_) (1)

where I_3_ and I_5_ are fluorescence intensities of Cy3 and Cy5, respectively, and coefficients 0.19 and 0.81 provide correction for the spectral cross-talk between the Cy3 and Cy5 detection channels. E_PR_ is FRET efficiency without correction for quantum yields of labels and an instrumentation factor. Sample sizes varied from 2000 to 4000 particles (nucleosomes or complexes) per measurement. The frequency distributions of nucleosomes based on E_PR_ (E_PR_ profiles) were plotted, averaged over at least three independent experiments, presented as mean ± SEM, and fitted by a sum of Gaussian peaks.

### 2.6. Electrophoretic Mobility Shift Assay (EMSA)

The probes were subjected to electrophoresis in 4% polyacrylamide gel (acrylamide: bisacrylamide 59:1; 0.2× TBE buffer). Fluorescent images of electrophoregrams were recorded using an Amersham Typhoon RGB laser scanner (Cytiva, Marlborough, MA, USA). Fluorescence was excited in gel at the 532 nm wavelength and detected in the 570–610 and 650–700 nm spectral ranges for Cy3 and Cy5, respectively.

### 2.7. Native Gel Electrophoresis of Proteins in Agarose

Native electrophoresis of H1 and its complexes with PARP1 was performed in the agarose LE2 for nucleic acid analysis (Helicon, Moscow, Russia). Agarose (2%) was suspended in 0.5× TBE buffer, heated for complete dissolution, and layered onto a flat plate. Fluorescently labeled H1.0 (10 nM) was incubated with PARP1 (40 or 80 nM) in low-adhesion tubes in TB150 buffer for 30 min at 25 °C. To induce PARylation, nucleosomes were pre-incubated with PARP1 for 15 min in TB150 buffer, mixed with NAD^+^ (5 µM), and incubated for 35 min. Afterward, H1.0 was added, and the mixture was incubated for 15 min. Electrophoresis was run at 90 V for 1 h at room temperature. Analysis of electrophoregrams was performed using the Amersham Typhoon RGB scanner. Fluorescence was excited in gel at the 633 nm wavelength and detected in the 650–700 nm spectral range.

### 2.8. Western Blotting

Nucleosomes (4 nM) were incubated with PARP1 (40 nM) in the absence or in the presence of NAD^+^ (5–100 µM) for 40 min. Then, samples were diluted with 5× buffer (10% SDS, 25% β-mercaptoethanol, 0.05% bromphenol blue, 300 mM Tris-HCl, pH 6.8), heated to 95 °C for 10 min, loaded to the 4–12% gradient gel (Mini-protean gels TGX, Bio-Rad, Hercules, CA, USA), and subjected to electrophoresis (140 V for 1 h) in Tris-glycine buffer (0.1% SDS, 192 mM glycine, 25 mM Tris-HCl, pH 8.6). Proteins were transferred to a nitrocellulose membrane (Bio-Rad) in the buffer containing 50 mM Tris-HCl, 50 mM MOPS, 3.5 mM SDS, 1 mM EDTA, and 20% ethanol (350 mA, 2 h, 4 °C). The membrane was further incubated for 1 h and washed twice with a solution containing 0.5% Tween-20, 37 mM NaCl, 2.7 mM KCl, 8 mM Na_2_HPO_4_, 2 mM KH_2_PO_4_, and 5% skimmed milk, stained with mouse monoclonal antibodies against polyADP-ribose (10H, ab14459, Abcam, Waltham, MA, USA) for 1 h, and next with horseradish peroxidase-conjugated secondary anti-mouse antibodies (Bio-Rad) for 1 h. The ChemiDoc system (Bio-Rad) and the SuperSignal™ West Pico PLUS chemiluminescent substrate (Thermo Fisher Scientific, Waltham, MA, USA) were used for image recording. Western blotting experiments were repeated three times.

### 2.9. Molecular Modeling

The HADDOCK 2.4 Web server [40,41] was used for molecular docking. The initial coordinates of the nucleosome:BRCT complex were taken from the 7scz structure [11], and the coordinates of the catalytic (CAT) domain from the 4dqy structure [42]. Residues were renumbered with pdb-tools [43] to conform to the input requirements of HADDOCK. Docking of CAT onto the nucleosome:BRCT complex was performed with the default parameters. Several nucleotide residues spanning the nucleosome dyad (chain I: 1–4, chain J: 0–2) were specified as “active” (i.e., definitely involved in interaction). These residues have been shown to interact directly with the H1 histone [36]. Amino acid residues on the surface of CAT (solvent accessibility > 40%) were specified as “passive” (i.e., possibly involved in interaction). Nucleosome:BRCT residues within 6.5 Å of the active residues were also specified as passive. To finalize the obtained docking model, 20 bp linkers were transferred from the 7k5x structure [44] and then optimized to remove clashes with the CAT domain. Optimization was performed using in-house Python scripts that implement algorithms of the 3DNA software package [45] together with a Monte Carlo modeling approach. In brief, a harmonic potential with a minimum at 7.5 nm was introduced between the first and last base pairs of the linker DNA region, and 10,000 random steps were performed in base-pair step coordinates to select an appropriate conformation based on DNA bending energy and steric clashes [46,47]. The selected DNA conformation was additionally optimized with Phenix [48].

## 3. Results

### 3.1. PARP1 Forms Three Types of Complexes with 2LN Nucleosomes and Reorganizes Nucleosome Structure

**Figure 2:**
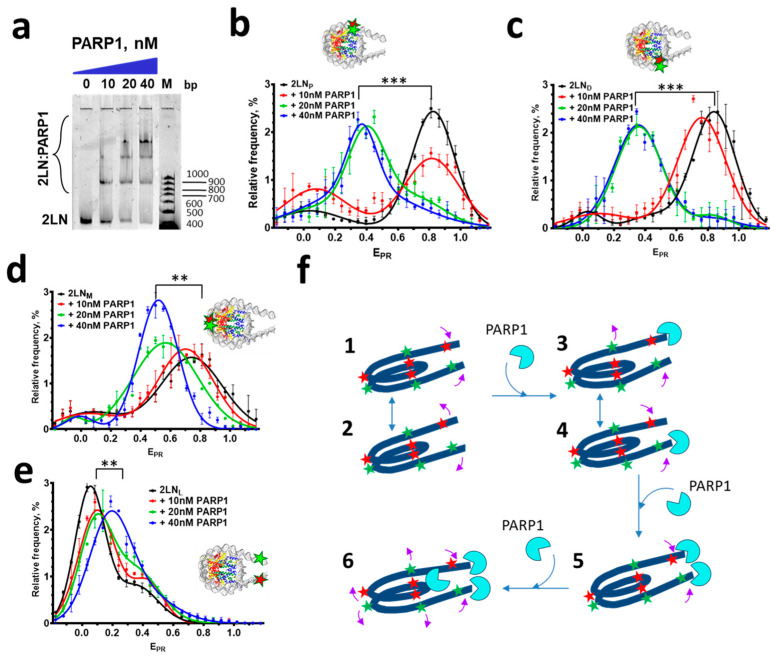
Formation of complexes of PARP1 with nucleosomes and concomitant rearrangement of nucleosome structure. (**a**) EMSA analysis of complexes of PARP1 with fluorescently labeled 2LN nucleosomes; M—DNA markers. (**b**–**e**) Analysis of PARP1 complexes with 2LN_P_ (**b**), 2LN_M_ (**c**), 2LN_D_ (**d**), and 2LN_L_ (**e**) nucleosomes using spFRET microscopy. Averaged E_PR_-profiles of nucleosomes are shown (mean ± SEM, number of experiments *n* = 3, 2000–4000 particles in each experiment). Changes in the position of the E_PR_ peak, *t*-test: *** *p* < 0.0005, ** *p* < 0.005. (**f**) Schematic model of interactions of PARP1 with 2LN nucleosomes. The nucleosome has at least two conformations of linker DNA helixes (states 1 and 2). The first PARP1 molecule interacts with one of the DNA ends (i.e., DSBs) of the nucleosome (complexes 3 and 4), slightly changing the conformation of nucleosomal and linker DNA. The second molecule of PARP1 interacts with the second end of DNA (complex 5). The third PARP1 molecule interacts with the nucleosome core and induces an increase in the distance between the gyres of nucleosomal DNA and a decrease in the distance between the helices of linker DNA (complex 6). Red and green asterisks mark the positions of the Cy3 and Cy5 labels. Pink arrows indicate structural changes observed as alterations in E_PR_ (i.e., the distance between the Cy3 and Cy5 labels) for differently labeled 2LN nucleosomes.

Extending our previous studies of interactions of PARP1 with core nucleosomes and nucleosomes having one fragment of linker DNA [28], we investigated complexes formed between PARP1 and nucleosomes with two fragments of linker DNA (2LN, Figure 1). EMSA analysis revealed concentration-dependent formation of three types of complexes between 2LN and PARP1 (Figure 2a). Appearance of the first 2LN-PARP1 complex was observed at the PARP1 concentration of 10 nM. The second and third complexes formed nearly simultaneously at 20–40 nM PARP1. A further increase in the concentration of PARP1 led to the aggregation of the 2LN-PARP1 complexes. As shown earlier, nucleosome-PARP1 complexes differ in the number of PARP1 molecules bound to one nucleosome [28]. Accordingly, PARP1 forms complexes with 2LN with the stoichiometry (PARP1:2LN) 1:1, 2:1, and 3:1 (Figure 2a,f).

Structural features of the 2LN-PARP1 complexes were studied by spFRET microscopy (Figure 2b–d,f). Analysis of the E_PR_ profiles of 2LN_P_, 2LN_M_, and 2LN_D_ nucleosomes labeled in the core region shows that they are characterized by the presence of two subpopulations, which are different in E_PR_: a major subpopulation with high FRET values and a minor subpopulation with low FRET values (Figure 2c–e). The high-E_PR_ peak corresponds to intact nucleosomes, in which Cy3 and Cy5 labels are localized close to each other as a result of wrapping of nucleosomal DNA on the histone octamer. The low-E_PR_ peak indicates the presence of nucleosomes with partially unwrapped and/or histone-free DNA.

Nucleosomes 2LN_L_ labeled in the linker DNA region are characterized by the major subpopulation of particles having low FRET (peak at E_PR_~0.05), where helices of linker DNA-containing sites labeled with Cy3 and Cy5 are far from each other, and the minor subpopulation having medium FRET (shoulder at E_PR_~0.35), where helices of linker DNA are close to each other (Figure 2e). These data suggest that the 20 bp helices of linker DNA can adopt at least two conformations. Similar two conformations of linker DNA helices were found by spFRET microscopy previously for nucleosomes having linker DNA helices of 40 bp in length [32].

Studies of structural changes in the 2LN-PARP1 complexes show that the binding of the first molecule of PARP1, which occurs at ~10 nM PARP1 according to EMSA, slightly increases the subpopulation of nucleosomes with converged helices of linker DNA (Figure 2e); enhances unwrapping of nucleosomal DNA from histone octamer at the beginning of NPS (involving at least 13 bp of nucleosomal DNA) in some nucleosomes (Figure 2b); and increases slightly the distance between DNA gyres both near the end of NPS (in the 57 and 135 bp region, Figure 2c) and in the 35 and 112 bp region of NPS (Figure 2d). Binding of the second and especially the third molecule of PARP1, which occurs according to EMSA at 20–40 nM PARP1, is accompanied by the convergence of linker DNA regions (as follows from the shift of low-E_PR_ peak to ~0.2, Figure 2e) and the significant increase in the distance between DNA gyres in the core region of nucleosomes (as follows from the appearance of peaks at E_PR_~0.4, Figure 2b,c) and E_PR_~0.5 (Figure 2d), thus affecting the entire nucleosomal DNA.

Considering the higher affinity of PARP1 to damaged DNA [42,49,50], two PARP1 molecules likely interact with the ends of nucleosomal DNA (Figure 2f). Such interaction definitely relates to the mechanism of recognition of DSBs in DNA and proceeds via zinc finger domains of PARP1 [6,42,51]. EMSA and spFRET data suggest that this mode of PARP1 binding only slightly affects the structure of the nucleosome.

Since PARP1 also has affinity to intact DNA [11] and intact nucleosomal DNA [5], the third molecule of PARP1 very likely binds to the nucleosome core (Figure 2f). The interaction of PARP1 with intact DNA in the nucleosome occurs via the BRCT domain [11]. Importantly, it is the binding of PARP1 to the nucleosome core that leads to the considerable structural rearrangements of nucleosomal DNA. This conclusion is supported by the data on the similar PARP1-induced structural rearrangements that were observed in core nucleosomes (nucleosomes without linker DNA) and in the nucleosomes having one helix of linker DNA [28].

### 3.2. Poly-ADP-Ribosylation Causes Differential Dissociation of PARP1 Molecules from 2LN-PARP1 Complexes

**Figure 3:**
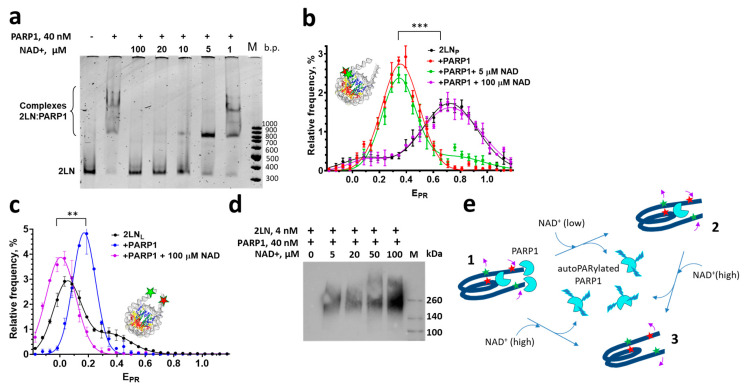
Effect of NAD^+^ on 2LN-PARP1 complexes. (**a**) EMSA analysis of the effect of different concentrations of NAD^+^ (1–100 µM) on the PARP1–nucleosome complexes. Concentration of PARP1 was 40 nM. The image was recorded using the fluorescence of fluorescently labeled nucleosomal DNA. (**b**) E_PR_ profiles of 2LN_P_ nucleosomes and their complexes with PARP1 (40 nM) in the absence and in the presence of 5 or 100 µM NAD^+^. (**c**) E_PR_ profiles of 2LN_L_ nucleosomes and their complexes with PARP1 (40 nM) in the absence and in the presence of NAD^+^ (100 µM). Inserts in (**b**,**c**) show the localization of fluorescent labels in the nucleosomes. Averaged E_PR_-profiles of nucleosomes are shown in (**b**,**c**) (mean ± SEM, number of experiments *n* = 3, 2000–4000 particles in each experiment). Changes in the position of the E_PR_ peak, *t*-test: *** *p* < 0.0005, ** *p* < 0.005. (**d**) Western blotting analysis of autoPARylation of PARP1 activated by 2LN nucleosomes at different concentrations of NAD^+^. (**e**) The model of the reorganization of PARP1–2LN complexes in the presence of low and high concentrations of NAD^+^. Red and green asterisks mark the positions of the Cy3 and Cy5 labels. Pink arrows indicate structural changes in 2LN nucleosomes observed as alterations in E_PR_ (i.e., the distance between the Cy3 and Cy5 labels) in comparison with intact nucleosomes.

The addition of NAD^+^ to the 2LN-PARP1 complexes activates the reaction of poly-ADP-ribose (PAR) synthesis that is accompanied by auto-poly-ADP-ribosylation (autoPARylation) of PARP1 (Figure 3d). As a result of the accumulation of negatively charged PAR chains on PARP1, all three types of 2LN-PARP1 complexes dissociate (Figure 3e, state 3), releasing intact nucleosomes, according to the EMSA data obtained at 100 μM NAD^+^ (Figure 3a). spFRET analysis confirms this and shows that the released nucleosomes recover their original structure in the core region (Figure 3e, state 3). This conclusion follows from the similarity of E_PR_ profiles of free 2LN and nucleosomes released from the complexes after addition of 100 μM NAD^+^ (Figure 3b). In the linker DNA region (Figure 3c), the conformation with a large distance between DNA helices dominates in the released nucleosomes, probably reflecting accumulation of additional negative charge on the nucleosome due to PARylation of core histones (Figure 3e, state 3). As shown earlier, the H2A and H2B histones can be PARylated in chromatin [52], although the PARylation of core histones occurs with much lower efficiency than autoPARylation [5].

At low concentrations of NAD^+^, such as 5 μM, PARylation occurs slowly, allowing the differential dissociation of PARP1 molecules from 2LN-PARP1 complexes to be observed. Two PARP1 molecules dissociate first (Figure 3e, complex 2), while the 1:1 2LN-PARP1 complex persists for a longer time, as revealed by EMSA (Figure 3a). In the 3:1 complex between PARP1 and 2LN, two PARP1 molecules form very similar complexes with the ends of linker DNA, while the nature of the complex formed by the third PARP1 molecule with the core region of the nucleosome is different (Figure 3e, complex 1). The ends of linker DNA (i.e., DSBs) provide conditions for the high catalytic activity of PARP1 molecules bound to these DNA “defects” and rapid auto-PARylation, followed by the dissociation of these complexes (Figure 3e, complex 2). Most likely, the complex of PARP1 bound to the core region of the nucleosome persists in the presence of NAD^+^ (Figure 3e, complex 2).

Due to the long lifetime of the preserved complex, its structural features can be characterized using spFRET microscopy. The E_PR_-profile of the 1:1 complex of 2LN_P_ with PARP1, which persists at 5 μM NAD^+^, is very similar to that of the 2LN_P_-PARP1 complex in the absence of NAD^+^ (Figure 3b). This indicates that the reorganized structure of the nucleosome is maintained in this 1:1 complex (Figure 3e, complex 2) and confirms our conclusion that the binding of PARP1 to the core region alters the nucleosome’s structure.

### 3.3. Can PARP1 Reorganize the Structure of a Chromatosome?

Considering that PARP1 reorganizes the nucleosome structure, the question arises as to whether it can also affect chromatosome structure. To address this question, the E_PR_ profiles of chromatosomes (complexes of 2LN_L_ with H1.0 histone) in the absence or presence of PARP1 were measured and compared with the E_PR_ profiles of 2LN_L_ nucleosomes and 2LN_L_-PARP1 complexes.

**Figure 4:**
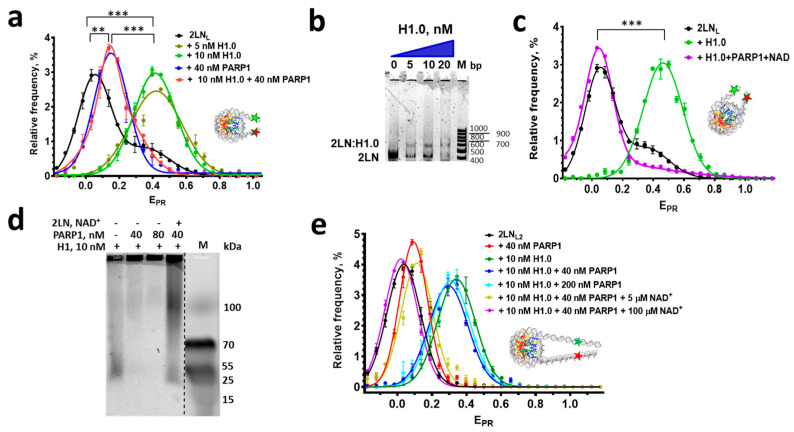
PARP1 binds H1 and can compete with H1 for binding to a nucleosome. (**a**) spFRET microscopy analysis of H1.0 complexes with 2LN_L_ nucleosomes and the competition between H1.0 and PARP1 for binding to 2LN_L_ nucleosomes. (**b**) EMSA analysis of 2LN nucleosomes and their complexes with H1.0. Image was recorded using fluorescence of labeled 2LN. (**c**) spFRET microscopy analysis of interactions between 2LN_L_, PARP1 (40 nM), and H1.0 (10 nM) in the presence of NAD+ (100 µM). (**d**) Native electrophoresis (2% agarose) of fluorescently labeled H1.0 in the absence or presence of PARP1 (40 or 80 nM), 2LN (4 nM), and NAD^+^ (100 µM). M—protein markers. Image was recorded using fluorescence of fluorescently labeled H1.0. (**e**) spFRET microscopy analysis of H1.0 complexes with 2LN_L2_ nucleosomes and the competition between H1.0 (10 nM) and PARP1 (40 or 200 nM) for binding to 2LN_L2_ nucleosomes in the absence of or in the presence of NAD^+^ (5 or 100 µM). (**a**,**c**,**e**) Averaged E_PR_ profiles are shown (mean ± SEM, number of experiments *n* = 3, 2000–4000 particles in each experiment). Changes in the position of the E_PR_ peak, *t*-test: *** *p* < 0.0005, ** *p* < 0.005.

According to the spFRET microscopy, the interaction between 2LN_L_ and linker histone H1.0 leads to the formation of a uniform population of complexes. This population is characterized by a single E_PR_ peak with a maximum at E_PR_ = 0.41 instead of the peak at E_PR_ = 0.05 and shoulder at E_PR_ = 0.35 in the E_PR_ profile of free 2LN_L_ nucleosomes (Figure 4a). These changes in the E_PR_ profile indicate a significant convergence of linker DNA helices induced by H1 (Figure S1a), which is known to contribute to the formation of higher-order chromatin structures [36,53,54,55]. As determined using the spFRET and EMSA analysis, a single predominant complex of H1.0 with nucleosomes forms at low-nanomolar concentrations of H1.0 (Figure 4a,b).

As discussed above, the binding of PARP1 to the nucleosome core also causes convergence of the linker DNA helices, which is accompanied by a significant shift in the low-E_PR_ peak from 0.05 ± 0.02 to 0.18 ± 0.02 (*p* < 0.005) in the E_PR_ profile of 2LN_L_ nucleosomes (Figure 4a). Thus, PARP1 and H1 induce qualitatively similar structural changes in the linker DNA region. At the same time, the convergence of the linker DNA helices in the 2LN_L_-PARP1 complex is less pronounced than in the case of the 2LN_L_-H1.0 chromatosome.

When PARP1 is added to 2LN_L_-H1.0 chromatosomes, the E_PR_ profile of the mixture becomes very similar to that of the 2LN_L_-PARP1 complex (Figure 4a), suggesting that PARP1 replaces H1.0 in the complexes with nucleosomes (Figure S1a). spFRET analysis also shows that the presence of PARylated PARP1 prevents the binding of linker histone H1 to the nucleosome: no characteristic convergence of linker DNA helices is observed in the reaction mixture containing nucleosomes, PARP1, NAD^+^, and H1.0 (Figure 4c). According to the data of native electrophoresis in agarose, H1.0 forms complexes with PARP1 and autoPARylated PARP1 (Figure 4d). This is manifested in disappearance of a signal from fluorescently labeled H1 on a gel, as complexes between PARP1 and H1.0 do not enter into the gel. In contrast, the complexes between fluorescent H1.0 and autoPARylated PARP1 appear as an extended band in the gel, since the autoPARylated PARP1 molecules have heterogeneous molecular masses and charges. The increased negative charge of these complexes appears to facilitate their migration through the gel. Some of H1.0 molecules can also be PARylated, and they produce a signal near the position of low-molecular-weight markers. Considering that in our experiments the concentration of PARP1 (40 nM) was higher than the concentrations of H1.0 (10 nM) and 2LN (1–3 nM), all H1.0 molecules are likely bound to PARP1 or autoPARylated PARP1 (Figure 4e). PARP1 molecules, which are in excess, could also bind to 2LN, whereas autoPARylated PARP1 did not form complexes with 2LN (Figure 4e). The binding of H1.0 to PARP1, but not to 2LN, suggests that the affinity of H1.0 to PARP1 and/or the affinity of PARP1 to the nucleosome are higher than the affinity of H1.0 to 2LN.

To verify the possible dependence of interactions between nucleosomes, H1.0 and PARP1 on the length of linker DNA, 2LN_L2_ nucleosomes were assembled, which contain 40 bp linker DNA fragments and Cy3 and Cy5 labels localized 25 bp before and after NPS (Figure 1). The E_PR_ profiles of these nucleosomes, their complexes with PARP1 and H1.0, differ in the E_PR_ peak position: 0.04, 0.09, and 0.34, respectively (Figure 4e).

The addition of PARP1 (40 nM and 200 nM) to the complex between 2LN_L2_ and H1.0 did not lead to considerable changes in the position of the E_PR_ peak (Figure 4e), indicating the preservation of the complex, likely complemented with the PARP1 binding to the ends of linker DNA (Figure S1b). It seems that the longer length of linker DNA (40 bp) dictates the higher affinity of H1.0 to 2LN_L2_ than to PARP1.

In contrast, the addition of PARP1 (40 nM) together with NAD^+^ to the 2LN_L2_-H1.0 complex led to changes in the E_PR_ profile of this complex. At 100 µM NAD^+^, the resulting E_PR_ profile resembled that of 2LN_L2_ (Figure 4e), which is consistent with the dissociation of the 2LN_L2_-H1.0 complex induced by the binding of autoPARylated PARP1 to H1.0 (Figure S1b). The activation of PARP1, which is required for PARylation, occurs through the binding of PARP1 to the ends of linker DNA. As discussed above, autoPARylated PARP1 loses its affinity for nucleosomes.

At 5 µM NAD^+^, the resulting E_PR_ profile is similar to that of the PARP1-2LN_L2_ complex (Figure 4e), which can be explained by slow PARylation (although it is sufficient for the dissociation of the 2LN_L2_-H1.0 complex caused by the binding of autoPARylated PARP1 to H1.0) and the formation of the PARP1-2LN_L2_ complex, where PARP1 is bound to the core region (Figure S1b).

### 3.4. Molecular Modeling of the PARP1 Binding to the Core Region of a Nucleosome near the Nucleosome Dyad

**Figure 5:**
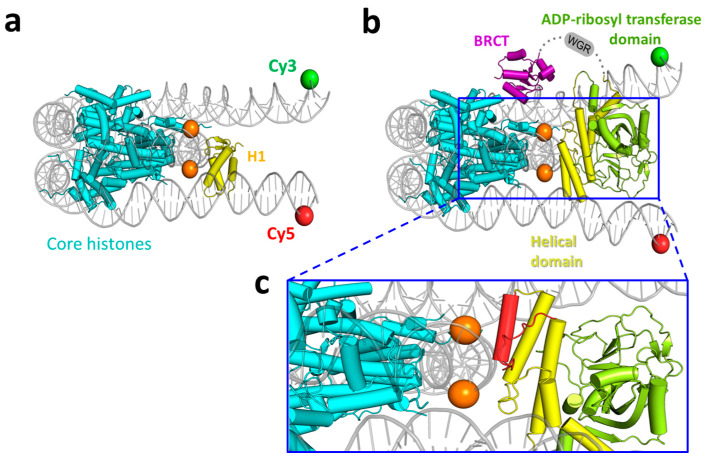
The molecular model of the complex between nucleosome and PARP1 (BRCT and CAT domains) and its comparison with the chromatosome structure. (**a**) CryoEM structure of chromatosome (7k5x). The core histones are colored cyan, DNA—gray, H1 histone—yellow. The dyad nucleosides are marked orange, and the positions of Cy3 and Cy5 labels in 2LN_L_—green and red, respectively. (**b**) The model of nucleosome:BRCT:CAT complex. The BRCT domain is shown in magenta, helical subdomain of CAT—yellow, and the ADP-ribosyl transferase subdomain—green. Other colors and notations are the same as in panel a. (**c**) Zoom of the model (**b**) in the area of interaction between the nucleosome and CAT domain. The interacting part of the helical subdomain (residues 725–754) is shown in red. Other colors and notations are the same as in panel b. The figure was prepared using PyMOL (http://pymol.org; accessed on 24 August 2025).

The binding of the BRCT domain of PARP1 to “superhelix location 7” on a nucleosome was identified [11], which is close to the site of H1 binding at the nucleosome dyad (Figure 5a,b). Our data show that (i) the PARP1 binding to the core region of a nucleosome causes a similar (though less significant) convergence of the linker DNA helices as H1.0 causes (Figure 4c), (ii) H1.0 cannot bind to the 2LN nucleosome in the presence of PARP1 (Figure 4c,d). Taken together, the data are consistent with the previous observations suggesting that there is an overlap between the binding sites of PARP1 and H1 on the nucleosome [11].

To evaluate this possibility, the binding mode of PARP1 to the core region near the nucleosome dyad was elucidated using molecular modeling experiments. The model was based on the cryoEM structure of a complex of a core nucleosome with the BRCT domain of PARP1 (PDB ID 7scz) and an X-ray structure of PARP1 (4dqy; contains Zn1, Zn3, WGR, and CAT domains bound to a DNA strand break). As these structures do not share common domains, a suitable model cannot be obtained by simply superimposing the multi-domain crystal structure of PARP1 on the nucleosome:BRCT complex. Furthermore, certain changes in the PARP1 domain organization may occur during the complex formation due to flexible linkers between PARP1 domains [11,56].

Creating the model, the following experimental data were considered: the binding of PARP1 to undamaged plasmid DNA is mediated by the BRCT domain and helical subdomain of the CAT domain, but not the WGR domain [11], and the isolated CAT domain retains the ability to bind to chromatin [57]. As the first step, we constructed the nucleosome complex with BRCT and CAT domains. Using the nucleosome:BRCT complex coordinates, the CAT domain of PARP1 was docked to the nucleosome dyad. Docking positions of CAT were subjected to filtration using the following criteria: (i) CAT should interact with the nucleosome body, and not the nucleosome ends; (ii) CAT should not occupy the position of extended DNA linkers, and (iii) the N terminus of CAT should be oriented towards the C terminus of BRCT, in accordance with the PARP1 sequence. After a suitable CAT orientation was selected, the coordinates of 20 bp linkers were transferred from the 7k5x chromatosome structure and optimized. The obtained nucleosome:BRCT:CAT model is shown in Figure 5b. From the comparison with the cryoEM structure of the chromatosome (7k5x, Figure 5a), it is clear that the helical subdomain of CAT occupies the H1 binding site at the nucleosome dyad.

The interaction between the helical subdomain of CAT and nucleosomal DNA occurs at the dyad region through the αE helix and adjacent loop (Figure 5c), which is in agreement with hydrogen–deuterium exchange mass spectrometry data [11]. The WGR domain transferred to the obtained model from the 4dqy structure of PARP1 clashes with DNA (Figure S2). Thus, WGR is likely localized above the nucleosome plane due to PARP1 conformational mobility and flexibility of linkers connecting WGR with the BRCT and CAT domains (Figure 5b), also in agreement with the experimental data [11].

## 4. Discussion

Up to three PARP1 molecules can interact with a nucleosome having two fragments of linker DNA (Figure 2): two molecules bind to the blunt ends of nucleosomal DNA and one directly to nucleosomal DNA, possibly via the BRCT and/or CAT domains [11]. The latter “non-classical” interaction reorganizes the structure of a nucleosome, and this reorganized structure is preserved even after the PARylation-induced dissociation of two PARP1 molecules that were bound to the DNA ends (Figure 3). Our data on the binding of just one PARP1 molecule to the core region of a nucleosome are consistent with the observation that the binding of PARP1 is saturated at a 1:1 molar ratio between PARP1 and nucleosomes assembled on a circular plasmid [5].

The dissociation constant for the complex between PARP1 and intact DNA in the core region of a nucleosome is of the same value (~10–40 nM) as for the complex between PARP1 and blunt ends of DNA (i.e., DSBs) (Figure 2a), and neither the absence of linker DNA fragments nor the presence of one or two linker DNA fragments significantly change the affinity of PARP1 for nucleosomes [28]. This conclusion and the value of affinity of PARP1 for the nucleosome are in line with those reported for a three-nucleosome array, both with and without linker DNA fragments [50]. At the same time, we clearly recognize the binding of up to three PARP1 molecules to a nucleosome, in contrast to the previous opinion about one PARP1 molecule binding to either a mononucleosome or a three-nucleosome array [50]. According to the published estimation, the affinity of PARP1 for histone-free, intact DNA is 1.55 μM [11]. Thus, the presence of core histones and/or topological features of nucleosomal DNA increase the affinity of PARP1 to nucleosomes by 50 times compared to that for histone-free, intact DNA. These data explain the results of the analysis of distribution of PARP1 in chromatin of *Drosophila* cells that revealed tight association of PARP1 with core histones in the completely digested chromatin [9]. Also, our data support the conclusion about the predominant association of PARP1 with nucleosomes and not with nucleosome-free DNA in chromatin [9]. In addition, DNA damage leads to the accumulation of PARP1 at the sites of DNA lesions, as reported earlier [58,59], in particular, due to the high affinity of PARP1 for DSBs in DNA.

At the high concentration of NAD^+^ (100 μM), autoPARylation of PARP1 leads to the dissociation of all PARP1-nucleosome complexes and the restoration of the intact conformation of nucleosomal DNA but with an increased distance between the helices of linker DNA in the released nucleosomes (Figure 3). In agreement with previous observations made on a circular nucleosome array [5], spFRET microscopy did not reveal the release of DNA from nucleosomes with or without linker DNA fragments (Figure 2 and Figure 3, and the data from [28]) regardless of the PARylation activity.

The long-term preservation of the 1:1 PARP1:nucleosome complex with a reorganized nucleosome structure was found at a moderate concentration of NAD^+^ (5 μM) (Figure 3), and PARP1 was concluded to bind to the core region of a nucleosome in this complex. The persistence of this complex under these conditions, which favor autoPARylation and dissociation of PARP1 from nucleosomes, might be due to the low or negligible catalytic activity of PARP1 bound to the core region. Indeed, PARP1 bound to intact DNA is not activated and cannot undergo autoPARylation [5,50]. In addition, PARylation activity of PARP1 was reported to be stimulated by nucleosomes formed on a circular plasmid, resulting in a level of autoPARylation that is even higher than that induced by damaged DNA [5]. In any case, in our experiment (Figure 3), the depletion of non-PARylated PARP1 in solution during its autoPARylation at the ends of DNA, followed by dissociation, should shift the equilibrium towards the dissociation of any PARP1 complexes with a nucleosome.

It is known that at low concentrations of NAD^+^, the rate of PARylation is low, a small number of PAR chains are formed, and they are short. As the concentration of NAD^+^ increases, the rate of PARylation increases, and long chains of PAR are synthesized [60]. Accordingly, it is possible that the low level of autoPARylation of PARP1 does not destabilize its complex with the core region due to the specific binding mode, in contrast to PARP1 bound to the blunt ends of DNA.

It was reported that linker histone H1 from calf thymus and PARP1 can compete for binding to chromatin, displacing each other when there is an excess of one or the other protein [5]. Our experiments with 2LN nucleosomes and linker histone H1.0 from *X. laevis* are consistent with these data (Figure 4a). It was proposed that the competition occurs due to the overlapping of binding sites for H1 and PARP1 on a nucleosome [5]. Our molecular modeling data suggest that if the position of the BRCT domain is similar to that in the complex with the nucleosome as observed by cryo-electron microscopy [11], the CAT domain may interact with the dyad, and the WGR domain should be positioned above the nucleosome plane (Figure 5b,c). This model is consistent with the data on PARP1-induced protection of restriction sites near the dyad axis of a nucleosome and near the exits of DNA from the nucleosome [5].

Another factor that contributes to this competition is the high-affinity direct interaction between H1.0 and PARP1 in solution (Figure 4b). When the protein, which is present in excess, binds all molecules of the competitor protein, the unbound molecules gain access to the binding site on the nucleosome core. The observed competition is also consistent with the observation that the affinity of H1.0 for the 2LN nucleosome is similar to that of PARP1 for the 2LN nucleosome (Figure 2 and Figure 4a,b). At the same time, our experiments show that the efficiency of this competition depends on the length of the linker DNA fragments (Figure 4). Longer linker DNA fragments (40 bp) drastically decrease the ability of PARP1 to displace H1.0 from the complex with the nucleosome, most likely due to the increase in the stability of the chromatosome containing longer linkers (Figure 4e and Figure S1b). It is possible that the ability of PARP1 to displace H1 from nucleosomes in chromatin is modulated by the density of nucleosomes on DNA. The displacement of H1 from closely spaced chromatosomes by PARP1 may reduce chromatin compaction locally and provide facilitated access of PARP1-dependent factors (including DNA repair and pioneer transcription factors) to nucleosomes and inter-nucleosome DNA, while their binding to nucleosomes, which were reorganized by PARP1, may be enhanced. In line with this suggestion, increased PARP1-dependent binding of pioneer factor Sox2 was observed in the chromatin regions with a high density of nucleosomes [13].

As follows from our data, chromatosomes that are spaced more distantly from each other are more resistant to PARP1 binding and are able to maintain a compact structure of transcriptionally inactive chromatin. The results of our experiments also suggest that a moderate concentration of NAD^+^ is sufficient to revert this resistance by activated PARP1 (Figure 4e,g), while the nuclear concentration of free NAD^+^, as estimated, can achieve 70 µM [61]. Indeed, there are various factors that modulate PARylation processes in vivo. Thus, PARylation is negatively regulated by poly(ADP-ribose) glycohydrolase [62] and ATP [5,63].

Intense PARylation promotes displacement of H1 from chromatosomes without the binding of highly autoPARylated PARP1 to vacant binding sites on nucleosomes (Figure 4c,e and Figure S1b). Usually, intense PARylation is the result of the appearance of multiple damages in DNA. In this case, the displacement of H1, accompanied by the decondensation of chromatin, may facilitate finding the damaged sites in DNA within the chromatin. When DNA lesions are located near or within the nucleosome core, H1 displacement seems to be necessary to remove steric obstacles for proper recruitment and functioning of DNA repair factors.

## Data Availability

The data presented in this study are available on request from the corresponding authors. The data are not publicly available due to local regulations.

## Supplementary Materials

The following supporting information can be downloaded at: https://www.mdpi.com/article/10.3390/cells14171309/s1, Figure S1. Models of interactions of H1 and PARP1 with 2LNL (a) and 2LNL2 (b) nucleosomes, having different lengths of linker DNA (20 bp and 40 bp, respectively); Figure S2: Clashes between the nucleosomal DNA (gray) and WGR domain (magenta) transferred from the 4dqy crystal structure of PARP1 to the model of nucleosome:BRCT:CAT complex shown in Figure 5.

## Abbreviations

BRCT	domain of PARP1 containing BRCA1 C terminus motif
CAT	catalytic domain of PARP1
Cy3 and Cy5	fluorescent dyes cyanine 3 and cyanine 5
DSBs	double-strand breaks
EMSA	electrophoretic mobility shift assay
E_PR_-	proximity ratio (FRET efficiency without correction for detection sensitivity and quantum yields of fluorophores).
FRET	Forster resonance energy transfer
PARP1	poly(ADP-ribose)polymerase 1
NAD^+^	β-nicotinamide adenine dinucleotide
NPS	nucleosome positioning sequence
spFRET	single particle Forster resonance energy transfer
2LN_P_	nucleosomes that were assembled on the 187 bp DNA template containing the 147 bp Widom 603-42A NPS flanked by a pair of 20 bp linkers. Cy3 and Cy5 labels were attached to thymine bases at positions 13 and 91 bp from the beginning of NPS
2LN_M_	nucleosomes that were assembled on the 187 bp DNA template containing the 147 bp Widom 603-42A NPS flanked by a pair of 20 bp linkers. Cy3 and Cy5 labels were attached to thymine bases at positions 35 and 112 bp from the beginning of NPS
2LN_D_	nucleosomes that were assembled on the 187 bp DNA template containing the 147 bp Widom 603-42A NPS flanked by a pair of 20 bp linkers. Cy3 and Cy5 labels were attached to thymine bases at positions 57 and 135 bp from the beginning of NPS.
2LN_L_	nucleosomes that were assembled on the 187 bp DNA template containing the 147 bp Widom 603-42A NPS flanked by a pair of 20 bp linkers. Cy3 and Cy5 labels were positioned in linker DNA fragments at a distance of 18 bp before and after NPS
2LN_L2_	nucleosomes that were assembled on the 227 bp DNA template containing the 147 bp Widom 603-42A NPS flanked by a pair of 40 bp linkers. Cy3 and Cy5 labels were positioned in linker DNA fragments at a distance of 25 bp before and after NPS
