# Supplementary material for: Non-Classical H1-like PARP1 Binding to Chromatosome
Source: Cells. 2025 Aug 25;14(17):1309. doi: 10.3390/cells14171309 (PMC12427767; doi:10.3390/cells14171309)
Supplement: Supplementary file 1 [file cells-14-01309-s001.zip › cells-3821937-supplementary.pdf]

Non-classical H1-like PARP1 binding to chromatosome

Darya Koshkina, Natalya Maluchenko, Dmitry Nilov, Alexander Lyubitelev, Anna Korovina, Sergey Pushkarev, Grigoriy Armeev, Mikhail Kirpichnikov, Vasily Studitsky, Alexey Feofanov

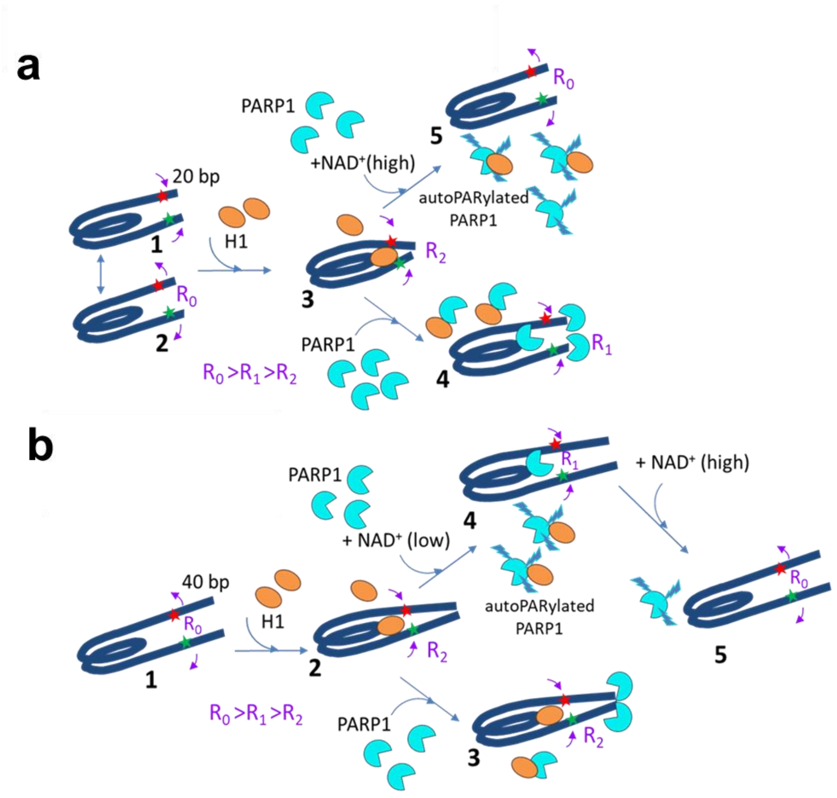

Figure S1. Models of interactions of H1 and PARP1 with 2LNL (a) and 2LNL2 (b) nucleosomes, having different lengths of linker DNA (20 bp and 40 bp, respectively). Red and green asterisks mark positions of Cy3 and Cy5 labels. R0, R1 and R2 are distances between helices of linker DNA at the labeled sites. Pink arrows indicate structural changes observed as alterations in EPR (i.e. the distance between the Cy3 and Cy5 labels) after binding of PARP1 to 2LNL and 2LNL2 nucleosomes.

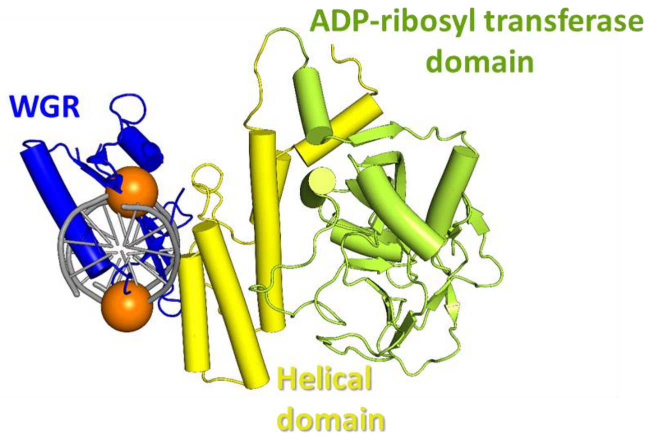

Figure S2. Clashes between the nucleosomal DNA (grey) and WGR domain (blue) transferred from the 4dqy crystal structure of PARP1 to the model of nucleosome:BRCT:CAT complex shown in figure 5b. Other designations as in figure 5b. In the model of the nucleosome-PARP1 complex, WGR cannot retain the same orientation relative to CAT as in the crystal of PARP1 (4dqy), and must occupy a region somewhere outside the nucleosome plane.
